# Honors to a Worthy Practitioner

**Published:** 1887-06

**Authors:** 


### HONORS TO A WORTHY PRACTITIONER.

PROFESSOR J. A. ROBISON writes: "I have just received the *Echo de Perse* from Teheran, Persia. It is published in French; and, among other things, contains the following:

'"We learn with great pleasure that by imperial firman His Majesty, the Shahinshah, has authorized the American missionaries to establish at Teheran a hospital, where, without regard to religion or nationality, all seeking relief shall be received for treatment.

"'Dr. W. W. Torrence, physician to the mission, has been appointed director of this establishment. His Imperial Majesty desiring at the same time to reward the zeal and devotion of Dr. Torrence, who for many years has been gratuitously relieving so much suffering and distress, has named him Grand Officer of the Order of the Lion and Sun of Persia.'

"Dr. Torrence's many friends will be gratified to hear of this high mark of distinction having been accorded him.

"Dr. Torrence was educated in Chicago, at Rush Medical College, graduating in 1880, and going to Persia in 1881. He has been laboring many years to establish a hospital there, but has had to contend against difficulties in raising funds."

